# Identification of a Novel Allosteric Inhibitory Site on Tryptophan Hydroxylase 1 Enabling Unprecedented Selectivity Over all Related Hydroxylases

**DOI:** 10.3389/fphar.2017.00240

**Published:** 2017-05-05

**Authors:** Mike Petrassi, Rob Barber, Celine Be, Sarah Beach, Brian Cox, Anne-Marie D’Souza, Nick Duggan, Martin Hussey, Roy Fox, Peter Hunt, Gabor Jarai, Takatoshi Kosaka, Paul Oakley, Viral Patel, Neil Press, David Rowlands, Clemens Scheufler, Oliver Schmidt, Honnappa Srinivas, Mary Turner, Rob Turner, John Westwick, Alison Wolfreys, Nuzhat Pathan, Simon Watson, Matthew Thomas

**Affiliations:** ^1^Genomics Institute of the Novartis Research Foundation, San DiegoCA, USA; ^2^Respiratory Disease Area, Novartis Institutes for BioMedical ResearchHorsham, UK; ^3^Novartis Institutes for BioMedical ResearchBasel, Switzerland; ^4^Translational Biology, Respiratory, Inflammation and Autoimmunity IMED, AstraZenecaGothenburg, Sweden

**Keywords:** serotonin, tryptophan, allosteric binding, pulmonary arterial hypertension

## Abstract

Pulmonary arterial hypertension (PAH) has demonstrated multi-serotonin receptor dependent pathologies, characterized by increased tone (5-HT_1B_ receptor) and complex lesions (SERT, 5-HT_1B_, 5-HT_2B_ receptors) of the pulmonary vasculature together with right ventricular hypertrophy, ischemia and fibrosis (5-HT_2B_ receptor). Selective inhibitors of individual signaling elements – SERT, 5-HT_2A_, 5HT_2B_, and combined 5-HT2_A/B_ receptors, have all been tested clinically and failed. Thus, inhibition of tryptophan hydroxylase 1 (TPH1), the rate limiting step in 5-HT synthesis, has been suggested as a more broad, and thereby more effective, mode of 5-HT inhibition. However, selectivity over non-pathogenic enzyme family members, TPH2, phenylalanine hydroxylase, and tyrosine hydroxylase has hampered therapeutic development. Here we describe the site/sequence, biochemical, and biophysical characterization of a novel allosteric site on TPH1 through which selectivity over TPH2 and related aromatic amino acid hydroxylases is achieved. We demonstrate the mechanism of action by which novel compounds selectively inhibit TPH1 using surface plasma resonance and enzyme competition assays with both tryptophan ligand and BH4 co-factor. We demonstrate 15-fold greater potency within a human carcinoid cell line versus the most potent known TPH1/2 non-specific inhibitor. Lastly, we detail a novel canine *in vivo* system utilized to determine effective biologic inhibition of newly synthesized 5-HT. These findings are the first to demonstrate TPH1-selective inhibition and may pave the way to a truly effective means to reduce pathologic 5-HT and thereby treat complex remodeling diseases such as PAH.

## Introduction

Serotonin (5-HT) has 15 receptors (5-HTR) divided into seven families, whose wide distribution and function both peripherally and within the central nervous system indicate the complexity of this signaling system (Berger et al., 2009). Despite this complexity, effective therapeutics of serotonin signaling have been developed; SERT antagonists for the treatment of depression (Stahl et al., 2013); 5-HT_1_ receptor isoform agonists for migrane headaches (Derry et al., 2014); 5-HT_2A_ receptor inhibitors as atypical anti-psychotics (Werner and Covenas, 2014); 5-HT_3_ receptor inhibitors for the treatment of chemotherapy-induced vomiting and irritable bowel syndrome (Cremonini et al., 2012). However, diseases characterized by more complex pathophysiology involving multiple elements of the 5-HT signaling cascade have failed to respond to selective receptor therapies.

Pulmonary arterial hypertension has demonstrated multi-serotonin receptor pathologies, characterized by increased tone (5-HT_1B_ receptor) and complex lesions (SERT, 5-HT_1B_, 5-HT_2B_ receptors) of the pulmonary vasculature together with right ventricular hypertrophy, ischemia and fibrosis (5-HT_2B_ receptor) (Thomas et al., 2013). Inhibitors of SERT (Shah et al., 2009), 5-HT_2A_ (Frishman et al., 1995), 5-HT_2B_ (Tomillero and Moral, 2008), and combined 5-HTR2_A/B_ (Dumitrascu et al., 2011) receptors have all been tested clinically and failed. Furthermore, carcinoid syndrome, in which carcinoid tumor metastases in the lung over-produce 5-HT (avoiding hepatic degradation) presents as a broad array of symptoms through several 5-HT receptors – nausea/vomiting, diarrhea (5-HT_3_ receptor), bronchoconstriction (5-HT_1B/2A_ receptors), right heart fibrosis (5-HT_2B_ receptor) (Druce et al., 2009). As with pulmonary endothelium in PAH, carcinoid tumors produce large amounts of 5-HT through the over-expression TPH1 – the rate limiting step in 5-HT synthesis (Lovenberg et al., 1967). Pre-clinical data in models of PAH using either siRNA blockade of TPH1 or therapeutic inhibition via the low molecular weight compound pCPA has shown profound effects on both vasoactive and remodeling disease indices (Morecroft et al., 2012). However, the therapeutic potential of pCPA is limited by additional activity on other aromatic amino acid hydroxylases (tyrosine and PH) and the alternative TPH isoform TPH2. The two TPH1 isoforms differ in distribution, with TPH1 responsible for peripheral 5-HT synthesis whereas TPH2 predominates in the brainstem, and is thus responsible for the majority of behavior-related 5-HT production and subsequent signaling (5-HT does not cross the blood brain barrier) (Walther et al., 2003). Early clinical data in carcinoid syndrome using pCPA revealed the liabilities of non-selective TPH inhibition, where TPH2 inhibition has been retrospectively attributed to psychosis observed in some patients (Engelman et al., 1967). More recent molecules such as Telotristat etiprate, which more potently inhibits TPH1/2 than pCPA, with better selectivity over related aromatic amino acid hydroxylases, have demonstrated efficacy in carcinoid syndrome trials showing some symptomatic improvements and good tolerability (Kulke et al., 2014). The distribution and activity of telotristat etiprate is predominantly gut restricted (Shi et al., 2008; Kim et al., 2015) - the principal site of physiologic TPH1 activity/5-HT production – avoiding the adverse effects of TPH2-mediated 5-HT production in the CNS and consequent psychiatric toxicity. However, the efficacy of such a therapeutic strategy is limited in diseases where the principal site of pathologic 5-HT production is distal to the gut, such as pulmonary endothelium in PAH or lung metastases in carcinoid syndrome.

Thus it is clear that there is a need for peripherally acting inhibitors of TPH1 with selectivity over TPH2. The high degree of homology between the isoforms (71% amino acid identity) and identical orthosteric site binding residues (Walther and Bader, 2003; Walther et al., 2003) make gaining selectivity an extreme medicinal chemistry challenge. Platelet storage of synthesized 5-HT also represents a challenge for biology in demonstrating inhibition of TPH1 activity *in vivo*. Here we describe the biochemical and biophysical characterization of a novel allosteric site on TPH1 through which selectivity over TPH2 is achieved and demonstrate the mechanism of action of compound inhibition. We describe the evolving potency and selectivity of TPH1-selective compounds, which originate from a novel high-throughput proximity assay screen. Furthermore, we detail novel *in vitro* and *in vivo* systems utilized to determine effective biologic inhibition of newly synthesized 5-HT by a novel antagonist series.

## Materials and Methods

### *In Vitro* TPH Activity Assays

Human TPH-1 and Human TPH-2 enzyme assays were carried out at room temperature with atmosphere oxygen in a volume of 25 μL. A base buffer of 40 mM HEPES pH 7.0, 200 mM ammonium sulfate was made and stored at 4°C. On the day of assay, final concentrations of 10 μM iron ammonium sulfate, 0.1 mg mL^-1^ BSA, 25 μg mL^-1^ catalase, and 0.04% Chaps were added and designated as enzyme buffer. Substrate buffer was made the same way but for the addition of a final concentration of 10 mM DTT. Enzymes were diluted as follows: 1.25x = 12.5 nM TPH1 (Final = 10 nM) or 1.25x = 37.5 nM TPH2 (Final = 30 nM) in enzyme buffer. Substrates were diluted as follows: 5x = 200 μM (6R)-5,6,7,8-Tetrahydrobiopterin dihydrochloride (BH_4_) (Final = 40 μM) and 5x = 100 μM Tryptophan (Final = 20 μM) in substrate buffer. Compounds were serially diluted 1:1 in DMSO to 50x = 500 μM (Final = 10 μM). Assays were performed in GNF Custom Greiner Black 384-well plates. Compounds were added at 0.5 μL per well. Enzymes were added at 20 μL per well. Compounds and enzymes were pre-incubated 15 min at room temperature. Substrates were added at 5 μL per well to initiate the reaction. The plates were covered and incubated at room temperature: 30 min for TPH1 and 60 min for TPH2. The reactions were quenched with the addition of 25 μL 30% sulfuric acid. The plates were read immediately with a PerkinElmer Envision reader at excitation = 280 and emission = 535.

### Protein Preparation and X-ray Crystallography

*Escherichia coli* BL21 Rosetta (DE3) cells harboring a plasmid encoding human TPH1 (amino acid residues 103–413 or residues 104–394) with N-terminal His6-tag were grown in a bio-reactor in auto induction medium. The constructs TPH1 (103–413) and TPH1 (104–394) were used for x-ray crystallization and SPR experiments, respectively (equivalent biochemical activity demonstrated – data not shown). Frozen cell pellets were homogenized in 50 mM Tris-HCl (pH 8.0), 400 mM NaCl, 1 mM tris(2-carboxyethyl)phosphine (TCEP), 5% glycerol, 0.1% 3-[(3-cholamidopropyl) dimethylammonio]-1-propanesulfonate and the protease inhibitor cocktail complete/EDTA free (Roche) and cells were lyzed with a microfluidizer. The protein was purified by Ni-NTA (Qiagen). The His_6_-tag was removed with HRV 3C protease cleavage followed by size exclusion chromatography (Superdex SPX200/16/60; GE Healthcare), using MES 25 mM, pH 6, NaCl 100 mM, Glycerol 5%, Methionine 3 mM, TCEP 2 mM as elution buffer. For crystallization purposes, the protein was concentrated to 10 mg ml^-1^. The resulting protein was estimated to be >95% pure and homogeneous by SDS-PAGE and reverse phase HPLC. The identity of the protein was further confirmed by N-terminal sequencing and mass spectrometry (Q-Tof, Micromass, Waters). Structural location diagrams were generated using Molsoft ICMPro modeling tool. Protein sequence alignment was enabled by RCSB protein databank and EMBL Cluster Omega analysis.

### Biacore

Biosensor experiments were performed on a Biacore T100 instrument (GE Healthcare). Biotinylation and capture of TPH1 was performed by adding a volume of freshly prepared EZ-link solution (dissolved in water) to an aliquot of TPH1(104–394) in a 2:1 molar ratio and incubated on ice. After 30 min of incubation, the reaction was stopped by the addition of 100 mM of Tris pH7.5. Unreacted biotin was removed by passing the solution over a fast desalting column (Thermo scientific #89849, equilibrated with PBS, 0.5 mM TCEP) three times. Biotinylated protein was aliquoted and kept at -80°C until use. Biotinylated TPH1 (104–394) was immobilized at 22°C on a SA chip surface (GE Healthcare) equilibrated with 10 mM Hepes, 150 mM NaCl, 1 mM TCEP, 0.05% Tween20, 5% glycerol, pH7.6. Approximately 3200RU were immobilized using this procedure. A streptavidin surface served as the reference.

Binding experiments were performed at 20°C using 10 mM Hepes, 150 mM NaCl, 1 mM TCEP, 0.05% Tween20, 5% glycerol, pH7.6, 2% DMSO as running buffer. To match precisely the DMSO concentration of the analytes and the running buffer all analytes were first prepared as a 100x stock in DMSO, then 1 ml analyte1 +1 ml analyte2 (or 1 ml DMSO) + 98 ml of 10 mM Hepes, 150 mM NaCl, 1 mM TCEP, 0.05% Tween20, 5% glycerol, pH7.6 were mixed to keep a final DMSO content of 2% in each sample. Using a flow rate of 50 ml min^-1^, samples were injected for 120 s and dissociation was monitored for 240 s. The data collection rate was 10 Hz. As a preliminary experiment, all analytes were run in a 8-point dose response mode under the conditions mentioned above to determine their affinity toward TPH1 (data not shown). Concentrations used for competition experiments were subsequently chosen around 5–10x the KD for each individual analyte (10 mM of NVS-TPH146 and 100 mM LP533401) and 2x the KD for L-Trp (100 mM). In supplementary studies investigating the relative binding characteristics versus BH2 co-factor and combination with L-Trp ligand, concentrations used were 5 mM for NVS-TPH146 and 100 mM for both BH2 and L-Trp (KD previously estimated to 11 and 56 mM, respectively, data not shown).

### Mode of Inhibition Studies

Mode of inhibition studies were performed in triplicate using the primary screen assay with varying concentrations of the substrate at fixed concentrations of inhibitor. For looking at the dependency of inhibition with tryptophan, BH4 was held at a fixed concentration of 10 μM and for looking at the dependency with BH4, tryptophan was held at a concentration of 10 μM. Reaction rates were monitored at fixed time points for up to 30 min and converted using a standard curve for 5-hydroxytryptophan. Slopes were subsequently calculated from the linear portions of the converted progress curves. The data was fitted globally to the equations describing competitive, non-competitive, mixed, and uncompetitive inhibition models (as described below) and best fit analysis was carried out using the Enzyme Kinetics module of SigmaPlot (V12.1).

Competitive (Full):

$$\begin{array}{c} v\;=\;\mathrm{Vmax}\;{\left(1+{\left(\mathrm{Km}\;S^{-}{}^{1}\right)}^{*}(1+I\;{\mathrm{Ki}}^{-}{}^{1})\right)}^{-}{}^{1} \end{array}$$

Competitive (Partial):

$$\begin{array}{c} v\;=\;\mathrm{Vmax}\;{\left(1+{\left(\mathrm{Km}\;S^{-}{}^{1}\right)}^{*}(1+I\;{Ki1}^{-}{}^{1}){\left(1+I\;{Ki2}^{-}{}^{1}\right)}^{-}{}^{1}\right)}^{-}{}^{1} \end{array}$$

Noncompetitive (Full):

$$\begin{array}{c} v\;=\;\mathrm{Vmax}\;{\left({\left(1+I\;{\mathrm{Ki}}^{-}{}^{1}\right)}^{*}(1+Km\;S^{-}{}^{1})\right)}^{-}{}^{1} \end{array}$$

Noncompetitive (Partial):

$$\begin{array}{c} v\;=\;\mathrm{Vmax}\;{\left({\left(1+Km\;S^{-1}\right)}^{*}(1+I\;{\mathrm{Ki}}^{-}{}^{1}){\left({1+I}^{*}\mathrm{beta}\;{\mathrm{Ki}}^{-}{}^{1}\right)}^{-}{}^{1}\right)}^{-}{}^{1} \end{array}$$

Mixed (Full):

$$\begin{array}{c} v\;=\;\mathrm{Vmax}\;{\left({\left(\mathrm{Km}\;S^{-}{}^{1}\right)}^{*}(1+I\;{\mathrm{Ki}}^{-}{}^{1})+(1+I\;{\left({\mathrm{alpha}}^{*}\mathrm{Ki}\right)}^{-}{}^{1})\right)}^{-}{}^{1} \end{array}$$

### Novel Cell Assay Assessment of TPH1 Activity

5-HT newly synthesized through TPH activity was assessed both *in vitro* and *in vivo* by addition of deuterium labeled substrate (deuterated tryptophan – TRP-d5) and subsequently generated deuterium labeled product (deuterated serotonin – 5HT-d4) (**Figure 6A**). BON (human pancreatic carcinoid) cell line was a gift from Dr. Courtney M. Townsend (University of Texas, Galveston, TX, USA) and cultured as previously described (Tran et al., 2004). Cells were seeded overnight in 96-well plates at 1.2 × 10^5^ cells per well in 200 μL growth media containing 1% FBS, and incubated at 37°C, 5% CO_2_. After 24 h, cell media was exchanged for 100 mL serum-free media. All reagents added to the cells in subsequent steps were diluted in serum-free media. Cells were treated with 25 μL of either compound or DMSO vehicle (1 % v/v) for 15 min at 37°C, 5% CO_2_ and then treated with 25 μL 196 mM deuterated-tryptophan (Isotec, UK) for a further 2 h at 37°C, 5% CO_2_. BON cell lysates were then prepared by removal of all media with subsequent addition of 250 mL HTAB. These lysates were further analyzed for D4-5-HT content by LCMS. Cell toxicity was also assessed in BON cells using a standard lactate dehydrogenase (LDH) assay according to instructions.

### Novel *In Vivo* Assessment of Pharmacokinetics/Harmcodynamics

*In vivo* inhibition of newly synthesized 5-HT was assessed by dosing dogs either acutely or chronically with compound followed by injection of TRP-d5 and subsequent 5-HT-d4 (versus compound) measurement in blood. Six male beagle dogs, 2.5–7.0 years of age and 8.6–11.0 kg weight were used in this novel, non-lethal model. The study was performed as a three-way, three-period crossover design with two animals assigned to one of three groups in turn (A–C in **Table 1**).

**Table 1:** Canine model design.

Group	Dosing: oral gavage, 0.5% MC : 0.5% (w/w) Tween 80, 4 mL kg^-1^, 5 h intervals		
A	veh	veh	veh
B	NVS-TPH120 100 mg kg^-1^	veh	veh
C	NVS-TPH120 100 mg kg^-1^	NVS-TPH120 100 mg kg^-1^	NVS-TPH120 100 mg kg^-1^

*Three-way, three-period crossover design with two animals assigned to one of three dose groups in turn.*

Three hours after the first dose TRP-d5 was injected subcutaneously at 50 mg kg^-1^ as a suspension in aqueous 0.5% methyl cellulose : 0.5% (w/w) Tween 80 at a volume of 1 ml kg^-1^. Approximately, 1 mL aliquots of EDTA blood was collected serially (0–100 h) from the vena cephalica and stored at –70°C for analysis. A 3-week washout period was applied before re-assignment of groups for the next period.

On the day of analysis blood samples were thawed and 50 μl aliquots were treated with 250 μl of acetonitrile containing the analytical internal standard (SDM) on a 96-well deep well plate for protein precipitation. The plate was shaken vigorously and the supernatant (200 μl) was separated by centrifugation, evaporated to dryness under nitrogen and the pellets were resuspended in 125 μl of water. Calibration standards were prepared in control dog blood and processed simultaneously with the study samples. The processed samples were analyzed by LC-MS/MS in a Sciex API 5500 mass spectrometer coupled with a Waters Acquity UPLC. The chromatography was employed on a Waters Atlantis dC18 analytical column, 100 mm × 2 mm, 3.5 μm at 50°C. The mobile phase A was aqueous 0.1% formic acid and the mobile phase B was methanol. The gradient program was as follows: 0–0.8 min 2% B, 0.8–1.8 min 2% to 90% B, 1.8–2.5 min 90% B, 2.5–2.6 min 90%–2% B. The flow rate was 0.5 ml min^-1^ and the sample injection volume was 5 μL. The mass spectrometer was operated in positive electrospray ionization mode and the analyte was detected using multiple reaction monitoring (MRM) transitions at m/z for 181 > 164 for 5HT-d4 and 311 > 165 for SDM. The synthetic 5HT-d4 with deuterium-labeling at different positions to the 5HT-d4 produced from TRP-d5 was used for the analytical reference due to their commercial availability. Because both forms of 5HT-d4 behaved almost identical on LC and were monitored using the same MRM transition for the loss of ammonia, the quality of the analytical results was considered appropriate. The analyte peak area was quantified against the calibration curves established in the same analytical run using Analyst 1.6 software. The lower limit of quantification was established at 0.4 ng ml^-1^.

For analysis of NVS-TPH120, samples were processed in a manner similar to that for 5HT-d4. After protein precipitation with acetonitrile the supernatant (250 μl) was separated by centrifugation and further diluted with 50 μL of water. The processed samples were analyzed by LC-MS/MS in a Sciex API 4000 mass spectrometer coupled with a Waters Acquity UPLC. The chromatography was employed on a Waters Acquity BEHdC18 analytical column, 50 mm × 2 mm, 1.7 μm at 50°C. The mobile phase A was aqueous 0.1% formic acid and the mobile phase B was methanol. The gradient program was as follows: 0-0.5 min 5% B, 0.5-1.0 min 5% to 95% B, 1.0–1.5 min 95% B, 1.5–1.6 min 95%–5% B. The flow rate was 0.6 ml min^-1^ and the sample injection volume was 10 μl. The mass spectrometer was operated in negative electrospray ionization mode and the analyte was detected using MRM transitions at m/z for 429 > 385 for NVS-TPH120 and 309 > 215 for SDM. The analyte peak areas were quantified against the calibration curve established in the same analytical run using Analyst 1.6 software. The lower limit of quantification was established at 46 nM.

Plasma protein binding of NVS-TPH120 in dog plasma was measured by equilibrium dialysis using RED device (Thermo). Plasma was 10-fold diluted with water and spiked with NVS-TPH120 at 5 μM for incubation. The obtained unbound fraction fu’ was converted to the corresponding unbound fraction fu in undiluted plasma using the formula fu = 1 (*D* ^∗^ (1 fu’^-1^ – 1 + 1 *D*^-1^))^-1^ where *D* is the dilution factor of 10.

### Animal Welfare and Ethics Statement

Animals used in these studies were housed and cared for throughout the experiment in accordance with Swiss Animal Welfare regulations (and in accordance to Directive 2010/63/EU) as they were performed in Switzerland. Study protocols were also assessed by the Novartis Institutes for Biomedical Research ethical review panel, to ensure conformation to the UK Animals (Scientific Procedures) Act 1986. Overnight fasting was applied before each experimental leg and food was returned 4 h after administration. All animals had free access to water before and throughout the experimental period. All procedures used were as humane as possible and complied with the recommendations of ARRIVE (Animal Research: Reporting In vivo Experiments).

### Data Analysis and Statistical Procedures

Biochemical, cell and *in vivo* data were analyzed via Graphpad Prism (version 5; La Jolla, CA, USA). Biosensor data were evaluated using Biaevaluation software (GE Healthcare). Mechanism of action analytics were carried out using the Enzyme Kinetics module of SigmaPlot (V12.1). Pharmacokinetic parameters were calculated by non-compartmental method using Phoenix (Pharsight). *In vitro* cell data and *in vivo* data are presented as mean ±SEM. *Ex vivo* 5HT-d4 concentrations in the NVS-TPH120-treated group were normalized for those in the control group at each time point in the same session. One-way ANOVA was used for comparison between groups followed by Dunnett *post hoc* test which compared each of the NVS-TPH120 dosed groups with the control group – data represented as a percentage reduction.

### Materials

Biochemical assay buffer reagents were purchased from Sigma–Aldrich (Poole, UK). Protein preparation reagents were purchased from Roche (Basel, Switzerland) and purification via Ni-NTA from Qiagen (Hilden, Germany). LDH cell toxicity assay was purchased from Pierce (Thermo Scientific). For Biacore experiments, L-Trp was purchased from Sigma–Aldrich (Poole, UK), BH2 was purchased from Schirks Laboratories (Jona, Switzerland). EZ-link Sulfo-NHS-LC-LC-Biotin was purchased from Fisher Scientific (Loughborough, UK). All other buffer reagents were purchased from Sigma–Aldrich (Poole, UK). Cell assay culture plates were purchased from Fisher Scientific (Loughborough, UK). Deuterated-tryptophan was purchased from Isotec (Southport, UK) and HTAB was purchased from Sigma-Aldrich (Poole, UK). L-tryptophan-2′,4′,5′,6′,7′-d5 (TRP-d5), methylcellulose, 1500 cP (MC), sulfadimethoxin from Sigma–Aldrich (Poole, UK) and serotonin-α, α,β,β-d4 (5HT-d4) from Qmx Laboratories (Dunmow, UK) were used for *in vivo* studies.

## Results

### Evolution in Potency and Selectivity of Allosteric TPH1 Inhibitors

**FIGURE 1:**
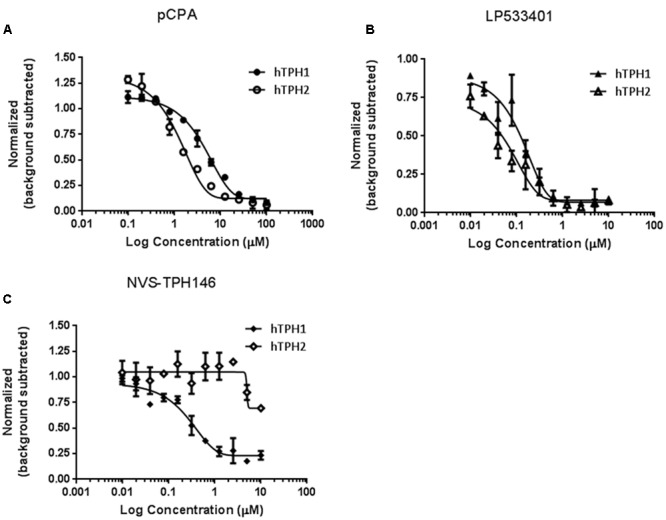
**Identification of selectivity for TPH1 over TPH2.** Compounds pCPA **(A)**, LP533401 **(B)** and NVS-TPH146 **(C)** were pre-incubated with hTPH1 and hTPH2 enzymes for 15 min. Reactions were initiated with the addition of the substrates BH4 and Tryptophan. The reactions were quenched with the addition of 30% Sulfuric Acid at 30 min for hTPH1 and 60 min for hTPH2. The plates were read immediately with a PerkinElmer Envision reader (excitation = 280, emission = 535).

Biochemical assays for TPH1 and TPH2 were used to assess increases in potency and selectivity for our emerging chemical series. Initially, dual TPH inhibitors were assessed for potency on both TPH1 and TPH2 in our assays. Relative IC_50_ values of 4.49 and 1.55 mM were revealed for pCPA on TPH1 vs TPH2. Values for the Lexicon next generation TPH inhibitor (LP533401) were 0.103 and 0.032 mM on TPH1 vs. TPH2, respectively. Selectivity was first observed in the original high throughput screen hit NVS-TPH146, with TPH1 : 2 ratio of 0.271 : 10 mM (**Table 2** and **Figures 1A–C**). Subsequent molecules (NVS-TPH176, -TPH180, and -TPH120) demonstrated incremental improvements in TPH1 potency (0.174, 0.039, and 0.021 mM) without erosion of TPH2 selectivity (>10 mM) (**Table 2**). Early, less potent examples within the chemical series were sufficiently potent to demonstrate selectivity and the novel binding mode. The advances in potency achieved with TPH120 enabled later *in vivo* experiments.

**Table 2:** Evolution of TPH1 inhibitors.

	TPH-1		TPH-2	
Compound	IC_50_ (μM)	SD	IC_50_ (μM)	SD
(1) pCPA	4.490	0.790	1.55	0.51
(2) LP533401	0.103	0.038	0.032	0.006
(3) NVS-TPH146	0.271	0.040	>10	n/a
(4) NVS-TPH176	0.174	0.038	>10	n/a
(5) NVS-TPH180	0.039	0.018	>10	n/a
(6) NVS-TPH120	0.021	0.009	>10	n/a

*Biochemical assay potencies on TPH-1 versus TPH-2. Examples 1–2 represent control orthosteric inhibitors. Examples 3–6 represent allosteric inhibitors of increasing TPH1 potency yet inactive on the TPH2 isoform.*

### Biophysical Characterization of the Allosteric Site

**FIGURE 2:**
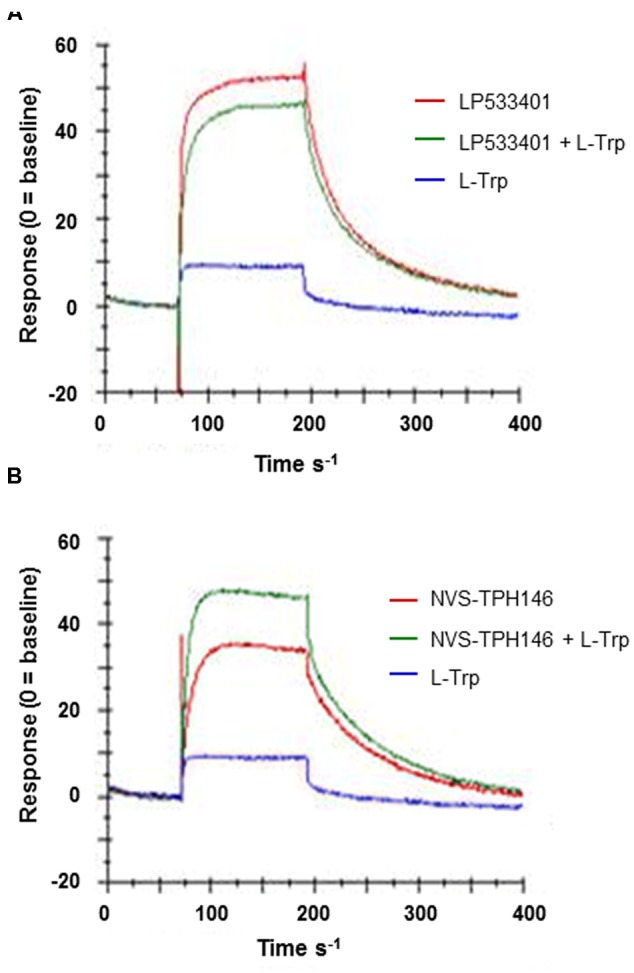
**Surface Plasma Resonance (SPR) sensorgrams for the interaction of TPH1 with various ligands.** Ligand (L-Tryp – blue) and compound (LP533401 or NVS-TPH146 – red) were injected singularly or in combination (green). Comparison of the resulting equilibrium responses between the individual or simultaneous injection allowed the classification of their binding mode for LP533401 **(A)** and NVS-TPH146 **(B)**.

**FIGURE 3:**
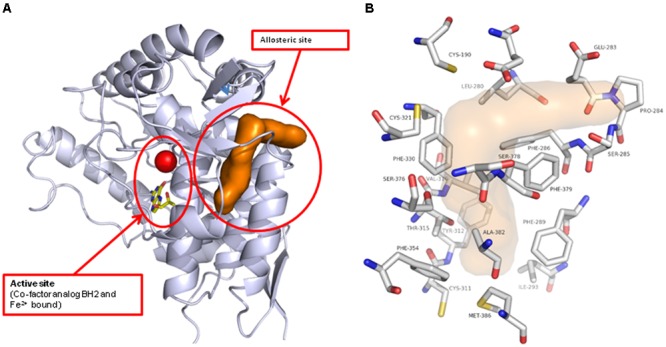
**Location of the novel TPH1 allosteric inhibitory site.** The TPH1 protein is shown in gray ribbon with the catalytic site Iron and cofactor shown. The allosteric site is indicated by the brown solvent accessible surface **(A)**. The TPH1 protein residues that form close contacts with the allosteric ligands are shown in atom-type colored capped sticks with the site indicated by the transparent surface **(B)**.

Do determine how and where the novel inhibitor class was binding, biophysical characterization of the site was performed. Surface plasma resonance (BiaCore) was used to confirm the allosteric versus orthosteric nature of NVS-TPH176 versus LP533401. The comparison of the resulting equilibrium responses between the individual or simultaneous injection of two ligands allows the classification of their binding mode. The obtained responses for the simultaneous injection of LP533401 and L-Trp is lower than the sum of individual injections. This suggests a competitive binding of both ligands (**Figure 2A**). The resulting equilibrium response for the simultaneous injection of NVS-TPH146 and L-Trp is comparable to the sum of the responses from single injections. This indicates that NVS-TPH146 and L-Trp bind simultaneously to TPH1 (**Figure 2B**). Comparable modes of inhibition for NVS-TPH146 were shown in studies co-injecting BH-2 co-factor and/or L-Trp (**Supplementary Figure S1**). The region of the novel allosteric site on TPH1 is shown in **Figure 3A** versus the orthosteric site where substrate, cofactor and Fe^2+^ bind. The residue interaction profile within the allosteric site is also revealed (**Figure 3B**). Sequence comparisons within the allosteric pocket show subtle differences between all isoforms (**Supplementary Figure S2**).

### Kinetic Analysis of Inhibition

**FIGURE 4:**
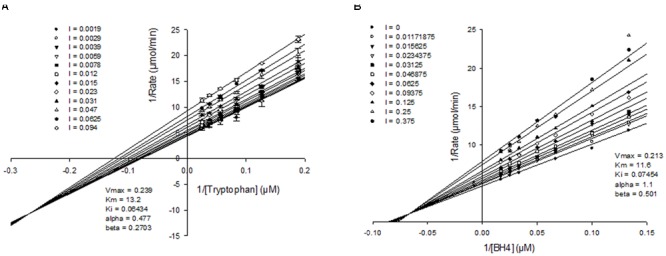
**Mechanism of action of allosteric inhibitors.** Double reciprocal plots of initial velocities at various concentration of the inhibitor NVS-TPH180 with varying **(A)** tryptophan concentration at a fixed non-saturating concentration of BH4 (10 μM) and **(B)**, varying BH4 at fixed non-saturating concentrations of tryptophan (10 μM). The inhibitor concentrations (l) used are as indicated in the graph and are represented in μM concentrations. Data is a mean ± SD from a single experiment, with each point in triplicate. Data was analyzed using Sigmaplot and fitted to a global fit analysis. Best fit anaylsis indicates a mixed partial inhibition with tryptophan with an α-value of 0.477 indicating a preference for the tryptophan bound complex.

To better understand how the novel class of molecules antagonized TPH1, kinetic analysis was carried out. Detailed mechanism of inhibition studies were carried out with purified truncated protein, by varying both tryptophan and BH4 concentrations in the presence of fixed concentrations of inhibitor. When tryptophan concentration was varied in the presence of a fixed sub-saturating concentration of BH4, NVS-TPH180 showed a mixed mode of inhibition indicated by the convergence of lines close or just above the x-axis on the double reciprocal plot when fitted globally using a non-linear regression fit based on partial mixed mode of inhibition for a single substrate inhibitor. NVS-TPH180 also demonstrated a strong preference for the tryptophan bound complex as indicated by its alpha factor being 0.273 (**Figure 4A**). Competition studies with BH4, using a sub-saturating concentration of tryptophan (10 μM), also shows that NVS-TPH180 displays characteristics of a mixed partial inhibitor when fitted globally using a non-linear regression fit based on partial mixed mode of inhibition for a single substrate inhibitor. The alpha value of 1.1 is also indicative of a small (non-significant) preference towards the free enzyme (**Figure 4B**). Re-plot methods looking at the dependence of Vmax and Vmax/Km (data not shown) against inhibitor concentrations also show very similar hyperbolic relationships re-confirming that this compound behaves in a mixed/ non-competitive manner with relative Ki values of 64 and 74 nM, and Km values of 13 and 11 μM, respectively, for both tryptophan and BH4 (**Figure 4**).

### Cell Activity of Allosteric TPH1 Inhibitors

**FIGURE 5:**
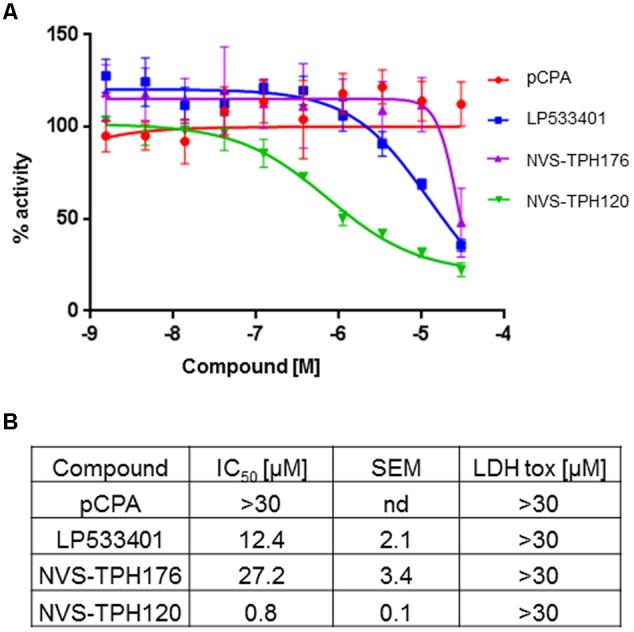
**BON (human carcinoid cell) cell responses to TPH1 inhibitors. (A)** Inhibition of D4-5-HT production. Compounds were incubated with BON cells for 15 min prior to addition of deuterated substrate. After 2 h intracellular D4-5-HT was extracted and quantified using LCMS/MS. **(B)** Table of compound efficacy vs. cell toxicity IC_50_ data. The data are expressed as means ± SEM; (*n* = 3).

Whole cell activity of TPH1 inhibition was assessed using BON cells (human carcinoid cell line) within a newly designed *in vitro* assay where new synthesiszed 5-HT could be detected via the labeling of tryptophan substrate with deuterium. LP533401 registered an IC_50_ of 12.4 mM, whereas a value for pCPA could not be established. The early allosteric inhibitor NVS-TPH176 registered 27.2 mM, whereas a >30-fold improvement in potency to 0.8 mM was observed with NVS-TPH120 (**Figure 5**). No impact on cell viability was detected for any compound up to 30 mM, assessed by LDH assay (**Figure 5B**).

### Inhibition of *De Novo* Synthesis of Serotonin by NVS-TPH120 *In Vivo*

**FIGURE 6:**
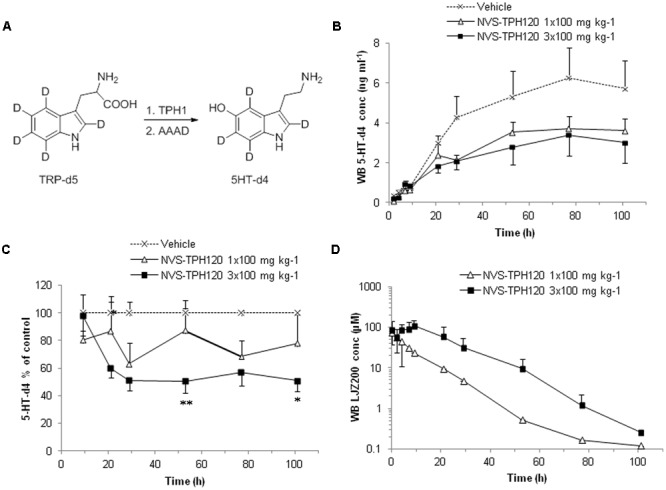
***In vivo* Inhibition of *de novo* synthesis of 5HT-d4 by NVS-TPH120.** The study was conducted as a 3-way crossover design. Male beagle dogs received orally vehicle or NVS-TPH120 2 h before and 3 h and 8 h after subcutaneous injection of TRP-d5 at 50 mg kg^-1^ (*n* = 6). Serially bled samples were analyzed by LC-MS/MS. **(A)** Metabolic pathway of TRP-d5 conversion to 5HT-d4. **(B)** Real values of blood 5-HT-d4 recovered from blood (baseline subtracted). **(C)** Blood 5HT-d4 levels % relative to the vehicle group. ^∗^*P* < 0.05; ^∗∗^*P* < 0.01. **(D)** Blood pharmacokinetic profiles of NVS-TPH120 (*n* = 6). AAAD: aromatic L-amino acid decarboxylase. The data are expressed as means ± SEM.

The degree to which *in vitro* inhibitory characteristics could be translated into animals, we developed an *in vivo* test system. Serotonin has long elimination half-lives in blood which hampers early and robust detection of reduced rate of serotonin synthesis. Furthermore, sequence and biochemical potency species cross-reactivity analysis indicated a drop-off in alignment in rodents (**Supplementary Figure S3**). To overcome this challenge, we developed a novel *in vivo* mechanistic model using a deuterium-labeled tryptophan and monitoring its metabolic conversion to the deuterated serotonin which can be discriminated from native serotonin by LC-MS/MS analysis (**Figure 6A**). NVS-TPH120 was dosed to dogs orally once or three times with 5 h intervals between the doses. TRP-d5 was injected s.c. 3 h after the first dose. In the 1 × 100 mg kg^-1^ group NVS-TPH120 blood concentrations reached a Cmax of 82 ± 38 μM at 2.3 ± 0.5 h (0.7 h before the TRP-d5 injection) followed by a monophasic decay at a mean elimination half-life of 7.7 ± 5.2 h (**Figure 6C**). Following 3 × 100 mg kg^-1^ dosing of NVS-TPH120 a peak concentration of 109 ± 40 mM was reached at 11.0 ± 1.1 h. The 3 × 100 mg kg^-1^ group achieved the mean AUC 3.3-fold higher than that after 1 × 100 mg kg^-1^ dosing. The synthesis of 5HT-d4 was inhibited in a dose-dependent manner although significant efficacy was not observed until 29 h (**Figure 6B**). The lowered level of 5HT-d4 was sustained up to 104 h after the first dosing of NVS-TPH120 in the 3 × 100 mg/kg group. The *in vitro* IC_50_ for the inhibition of dog TPH1 by NVS-TPH120 was scaled to the corresponding whole blood IC_50_ taking into account plasma protein binding. Due to very high plasma protein binding (fu = 0.0018) the corrected IC_50_ for blood was increased to 23 μM. The NVS-TPH120 concentration was maintained above the scaled IC_50_ up to 12 h post compound dosing at 1 × 100 mg kg^-1^ while the 3 × 100 mg kg^-1^ treatment exceeded this blood concentration for up to 32 h (**Figure 6D**).

## Discussion and Conclusion

Knockout studies have demonstrated the distribution of TPH isoforms in the body – TPH1 primarily expressed in the pineal gland and enterochromaffin cells of the gut, whereas TPH2 is restricted to neuronal cells (Cote et al., 2003; Walther et al., 2003; Patel et al., 2004). A breakthrough in pharmacologic inhibition of TPH was achieved with the discovery of molecules whose expanded pCPA core gained selectivity over related amino acid hydroxylases (phenylalanine and TH). Selectivity over TPH2 was effectively achieved through restricted distribution to the gut, including exclusion of enteric nerves (Shi et al., 2008; Margolis et al., 2014), providing both pre-clinical and clinical evidence for promising therapeutic options for gut-related disorders such as non-constipating irritable bowel syndrome (Brown et al., 2011; Kim et al., 2015). However, diseases in which pathogenesis is driven by TPH1 over expression, and subsequent 5-HT production, in peripheral tissues remain beyond the reach of these next generation inhibitors. Effective TPH1 inhibition beyond the gut requires pharmacologic selectivity over TPH2, and therefore a different chemical strategy entirely.

The TPH1-specific allosteric binding site described in these studies relies both on the robust, inventive nature of the assay systems used to investigate this novel biology, and on the availability of well characterized tool compounds for comparison. Literature characterization of pCPA and LP533401 have measured TPH1 activity at 250 and 0.7 mM IC_50_ values, respectively, (Liu et al., 2008). These differ from the values reported here by approximately 50- and 7-fold, respectively, revealing a higher degree of sensitivity to inhibition within the biochemical assay system described in each study. A 17-fold drop in potency is observed in the BON cell assay for LP533401 (pCPA effect not measurable above 30 mM without apparent toxicity). These data contrast previous reports which suggest a greater potency in the rat mastocytoma line RBL-2H3 than that seen biochemically – surprising discrepancies which may be based upon differing species and/or undetected/mild cell toxicity (Liu et al., 2008). Early allosteric inhibitors showed a biochemical and cell profile superior to pCPA, yet inferior to LP533401. The inhibitory potential of the allosteric pocket was only revealed in later examples (e.g., NVS-TPH120) which demonstrated 13- and 15-fold improvements over LP533401 in biochemical and cell potencies, respectively.

LP533401 was evolved from a common pCPA core, and thus the demonstration of an equivalent tryptophan substrate binding site could be anticipated and was confirmed in our BiaCore experiments (Cianchetta et al., 2010). The apparent chemical diversity of early inhibitor examples identified within our biochemical screen opened the possibility of a different binding mode. This hypothesis was supported by biochemical selectivity over TPH2 ->37-fold with the initial NVS-TPH146 compound, improving to >476-fold with NVS-TPH120. BiaCore studies provided the first real evidence of a novel allosteric binding site, when NVS-TPH146 and tryptophan ligand/co-factor were able to bind simultaneously – data further confirmed by the resolution of co-crystals containing NVS-TPH176 (data not shown due to proprietary nature of compound structure). The location of the allosteric site suggests the potential to influence orthosteric binding from the opposite side of the protein, as indicated by a common sequence of alpha helix (**Supplementary Figure S2**). Though there are subtle sequence differences in the allosteric site between each isoform, the exact mechanism by which selectivity for TPH1 is obtained with the novel inhibitors will likely require dynamic analytics to reveal. Mechanism of action experiments further demonstrated the lack of competitive binding to the substrate site, yet also suggested a preference for the tryptophan bound complex as indicated by its alpha factor being 0.273. This was reconfirmed by using re-plot methods in which we observed a larger titration in our Vmax versus inhibitor concentration plot as compared to our Vmax/Km plots - indicating that the inhibitor, although able to bind free enzyme at a low affinity, has a much higher preference and affinity for the substrate bound complex. This is consistent with X-ray data that shows the inhibitor binding to an area distal from the substrate binding site and suggests that this allosteric site becomes more accessible once the tryptophan binds. The alpha value of 1.1 for BH4 co-factor is also indicative of a small (non-significant) preference towards the free enzyme. Again, re-plot methods looking at the dependence of Vmax and Vmax/Km against inhibitor concentrations also show very similar hyperbolic relationships re-confirming that NVS-TPH180 behaves in a way entirely consistent with the X-ray observations that show a binding site distal to where BH4 resides. In both double-reciprocal plots, a beta value of less than one is generated, which indicates partial inhibition. However, it must be recognized that these assays are closed systems and as such one reasons for apparent partiality could be due to the time for the right complex to form, to allow the inhibitor to exert its full affect. Once it forms the final complex, the inhibitor does not come off until both substrates come off, also contributing to the final affect. Further evidence of partiality is *in vivo*, where inhibition of newly synthesized 5-HT plateaus at approximately 50% (**Figure 6**).

Deuterium-labeled tryptophan was also used *in vivo* as a novel method by which specifically new synthesized 5-HT could be monitored. The system was successfully validated in rat, rabbit and dog using pCPA as a reference TPH inhibitor (data not shown). Comparative species homology of TPH1 decreases from human to rat – differences which impacted the degree to which allosteric molecules inhibited TPH1 – demonstrated by NVS-TPH120 within biochemical assays with different species proteins (**Supplementary Figure S3**). Comparable potency and efficacy was observed between human and dog proteins with NVS-TPH120 and thus further *in vivo* studies used dogs. TPH1 inhibited via the allosteric site showed significant efficacy, peaking at 50%, which was sustained up to 100 h post tryptophan injection. Significant inhibition was not observed until 29 h as reflected by the delay in the increase of blood 5HT-d4 levels.

The next steps in the evolution of this chemotype of allosteric inhibitors is the resolution of eroded rodent cross reactivity and high plasma protein binding. Improvements in these areas would provide a tool by which more direct assessment of efficacy upon 5-HT-mediated complex remodeling diseases of the periphery could be made – an example of which could be the rodent hypoxia/SUGEN model of PAH which demonstrates increased TPH1 expression and 5-HT production, and is also sensitive to pCPA inhibition (Ciuclan et al., 2013; Thomas et al., 2013). Increased pulmonary artery endothelial cell (PAEC) TPH1 expression in PAH patients together with increased SERT expression in pulmonary artery smooth muscle cells (PASMC), may induce the hyperplasia central to PAH (Eddahibi et al., 2006). TPH1 knockout animals are protected from chronic hypoxia-induced pulmonary vascular remodeling and hypertension, although RV indices remain uninfluenced (Morecroft et al., 2007). PAEC:PASMC communication via 5-HT may not necessarily be paracrine, as demonstrated by co-culture studies investigating the role of 5-HT and TGFb signaling through myoendothelial gap junctions to effect PASMC differentiation (Gairhe et al., 2012). The pathologic disease relevance of PAEC-derived 5-HT was further investigated in selective *in vivo* gene therapy studies. Adenoviral vectors targeted to PAECs (utilizing bispecific antibody to angiotensin-converting enzyme (ACE) as the selective targeting system) were used to deliver small hairpin Tph1 RNA sequences in rats. Hypoxic rats increased lung Tph1 expression leading to the development of PAH pathologies, both of which were attenuated by PAEC-Tph1 gene knockdown (Morecroft et al., 2012). A recent publication by Abid and colleagues suggested combined gut and lung inhibition with LP533401. It is conceivable that a profound reduction in the whole body burden of 5-HT may impact peripheral pathology as the primary source of 5-HT is the gut (>95% in healthy individuals) which is then stored in platelets in circulation (Walther et al., 2003). However, the hypothesized inhibition of lung TPH1 by LP533401 was based on lung measures containing 5-HT-devoid platelets. In addition, the surprising lack of pathway activation within the chronic hypoxia model used questions the validity of serotonin pathway involvement / analytics (Abid et al., 2012).

The clinical applicability of effective, selective TPH1 inhibition in peripherial tissues beyond the gut does not end with PAH. Recent transcripome analysis has found an association between Bmpr1a deletion in mice and increased TPH1 expression in pancreatic islets, revealing a potentially important role for pancreatic 5-HT production in β-cell insulin production and glucose homeostasis (Jiang et al., 2015). These data are supported by studies in which Tph1-deficient mice fed a high-fat diet are protected from obesity, insulin resistance and nonalcoholic fatty liver disease while exhibiting greater energy expenditure by brown adipose tissue (Crane et al., 2015). Mechanistic evidence for 5-HT production, storage and release by T cells (particularly CD8^+^) is emerging which may bear relevance to an array of immune functional consequences in either health or disease (Chen et al., 2015). Furthermore, mast cell expression of TPH1, release of 5-HT and subsequent bronchoconstriction has been demonstrated in mice responding to allergen and IL-33, suggesting a pathologic role in the lung tissue of allergic asthmatics (Ahern, 2011; Sjöberg et al., 2015).

Thus, emerging evidence supports a role for TPH1 at the site of pathogenesis in complex remodeling, metabolic or immune diseases of peripheral tissues, and therefore necessitates a safe means of inhibition for effective therapy beyond the gut, yet without unwanted CNS effects. Here we describe a novel binding site on TPH1 to which the first molecules to achieve selectivity over TPH2 bind. The novel biologic systems generated to study these inhibitors also provide confidence that a major step forward has been taken in the development of a new class of safe peripheral serotonergic drugs.

## Author Contributions

CB, SB, A-MD, ND, MH, RF, PH, TK, PO, DR, CS, SH, MT, and RT made substantial contributions to the acquisition and analysis of data for the work. MP, NP, SW, MT made substantial contributions to the conception or design of the work. MP, RB, BC, GJ, NP, OS, JW, AW, NP, SW, MT made substantial contributions to the interpretation of data for the work. MT wrote the paper yet all authors were involved in drafting the work and revising it critically for important intellectual content. All authors approve the final version and agree to be accountable for all aspects of the work in ensuring that questions related to the accuracy or integrity of any part of the work are appropriately investigated and resolved.

## Conflict of Interest Statement

The authors declare that the research was conducted in the absence of any commercial or financial relationships that could be construed as a potential conflict of interest. The research conducted details preclinical drug discovery activities which were ultimately aimed at generating a novel therapeutic for the treatment of remodeling conditions dependent on serotonin dysregulation (e.g., pulmonary arterial hypertension). This research was funded by Novartis Institutes for Biomedical Research and formed part of a strategic initiative to develop an effective medicine, thereby associated/aligned with commercial needs.

## Supplementary Material

FIGURE S1 **Kinetic analysis of inhibitors binding to TPH1 (A)** Determination of the kinetic parameters of NVS-TPH146 binding to TPH1 **(B)** Determination of the kinetic parameters of LP533401 binding to TPH1 **(C)** Equilibrium response for the individual versus simultaneous (blue) injection of BH2 (red) and L-Trp (green) is comparable to the sum of the responses from single injections. **(D)** Equilibrium response for the individual or simultaneous (blue) injection of NVS-TPH146 (green) or BH2 + L-Trp (red).

FIGURE S2 **Sequence alignment of amino acid hydroxylases TH, PH, TPH1, and TPH2.** RCSB protein databank was used to obtain the human protein structures and sequences and EMBL for their Cluster Omega multi sequence alignment. Regions of the orthosteric site are shown in green and the allosteric site in blue, based upon residue interaction points (shown **Figure 3**).

FIGURE S3 **Percentage sequence homology table between species for TPH1 (A)**. Activity of NVS-TPH120 across the TPH1 assay using protein from different species **(B)**. Reactions were initiated with the addition of the substrates BH4 and Tryptophan. The reactions were quenched with the addition of 30% Sulfuric Acid at 30 min for hTPH1 and 60 min for hTPH2. The plates were read immediately with a PerkinElmer Envision reader (excitation = 280, emission = 535).

## Abbreviations

5-HT: 5-hydroxytryptamine
BH4: tetrahydrobiopterin
HTAB: hexadecyltrimethylammonium bromide
PAH: pulmonary arterial hypertension
pCPA: *p*-chlorophenylalanine
PH: phenylalanine hydroxylase
SERT: serotonin transporter
TPH: tryptophan hydroxylase
TH: tyrosine hydroxylase
