# Supplementary figures and images for: Identification of a Novel Allosteric Inhibitory Site on Tryptophan Hydroxylase 1 Enabling Unprecedented Selectivity Over all Related Hydroxylases
Source: Front Pharmacol. 2017 May 5;8:240. doi: 10.3389/fphar.2017.00240 (PMC5418348; doi:10.3389/fphar.2017.00240)

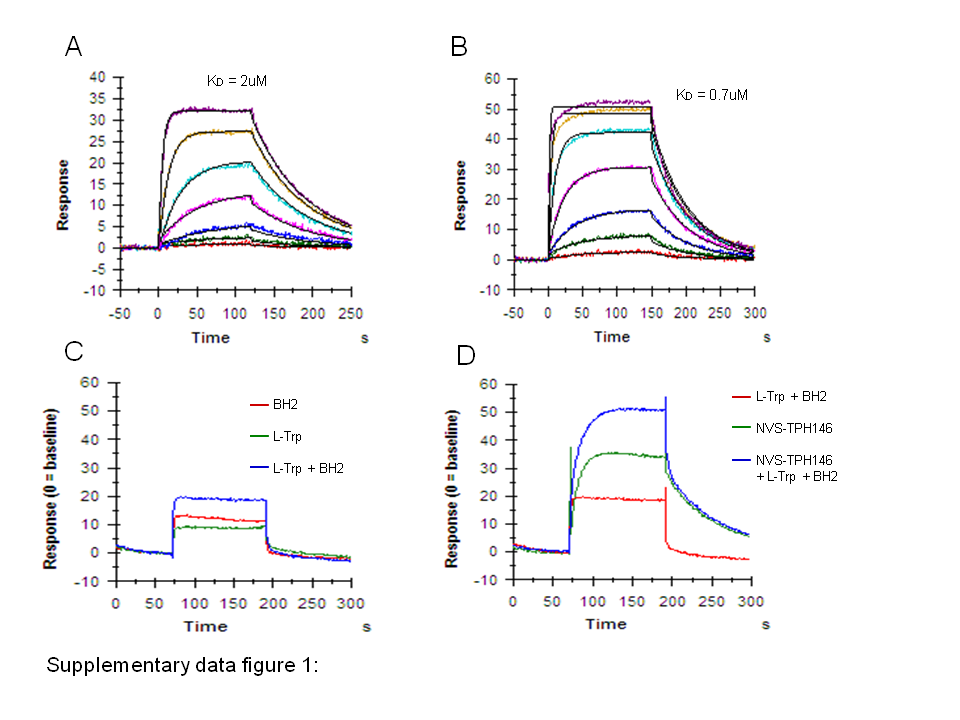

Supplement: FIGURE S1 — Kinetic analysis of inhibitors binding to TPH1 (A) Determination of the kinetic parameters of NVS-TPH146 binding to TPH1 (B) Determination of the kinetic parameters of LP533401 binding to TPH1 (C) Equilibrium response for the individual versus simultaneous (blue) injection of BH2 (red) and L-Trp (green) is comparable to the sum of the responses from single injections. (D) Equilibrium response for the individual or simultaneous (blue) injection of NVS-TPH146 (green) or BH2 + L-Trp (red). [file Image_1.TIF]

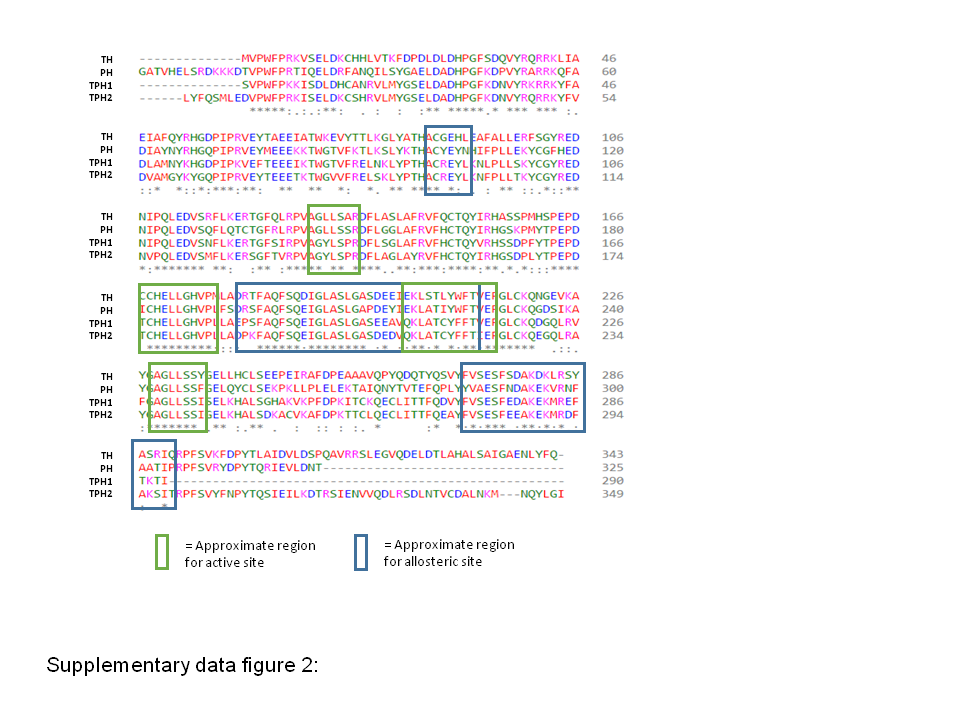

Supplement: FIGURE S2 — Sequence alignment of amino acid hydroxylases TH, PH, TPH1, and TPH2. RCSB protein databank was used to obtain the human protein structures and sequences and EMBL for their Cluster Omega multi sequence alignment. Regions of the orthosteric site are shown in green and the allosteric site in blue, based upon residue interaction points (shown Figure 3). [file Image_2.TIF]

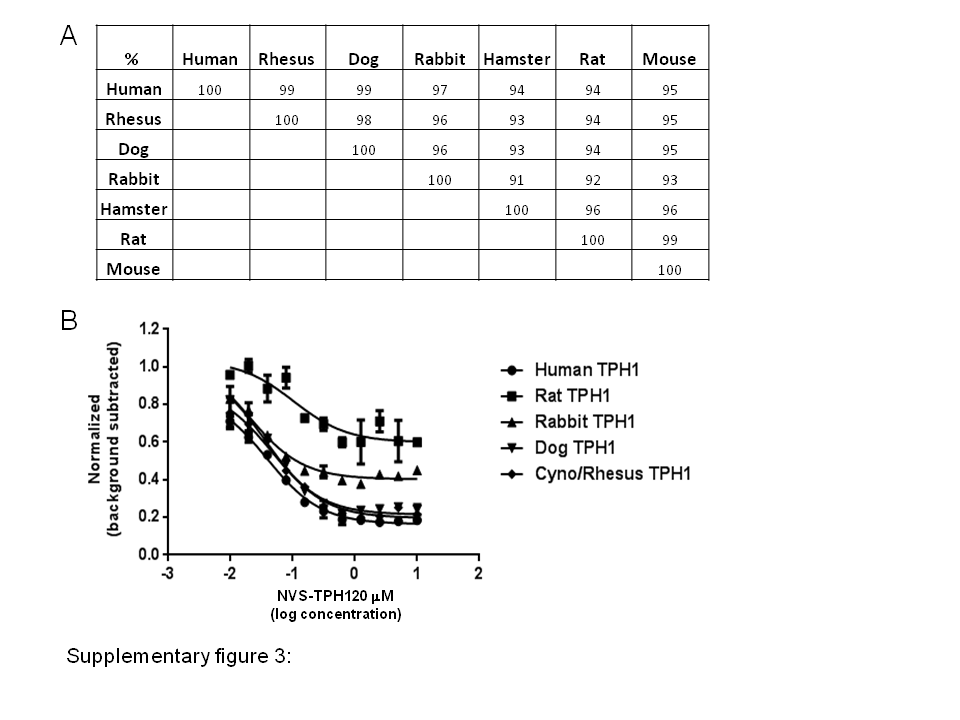

Supplement: FIGURE S3 — Percentage sequence homology table between species for TPH1 (A). Activity of NVS-TPH120 across the TPH1 assay using protein from different species (B). Reactions were initiated with the addition of the substrates BH4 and Tryptophan. The reactions were quenched with the addition of 30% Sulfuric Acid at 30 min for hTPH1 and 60 min for hTPH2. The plates were read immediately with a PerkinElmer Envision reader (excitation = 280, emission = 535). [file Image_3.TIF]
